# Nontraumatic Spondylolisthesis of the Axis with Cervical Kyphosis

**DOI:** 10.1155/2020/6859474

**Published:** 2020-03-19

**Authors:** Shuhei Mizobuchi, Nobuaki Tadokoro, Shogo Takaya, Katsuhito Kiyasu, Ryuichi Takemasa, Masahiko Ikeuchi

**Affiliations:** Department of Orthopaedic Surgery, Kochi Medical School, Kochi University, Kohasu, Oko-cho, Nankoku 783-8505, Japan

## Abstract

This study aimed at presenting a rare nontraumatic spondylolisthesis of the axis and considering its possible cause. Traumatic spondylolisthesis of the axis, called hangman's fracture, frequently occurs as a high-energy trauma. However, nontraumatic spondylolisthesis of the axis is quite rare, and relevant literature on this condition is scarce. We reported a case of a 49-year-old man who had spondylolisthesis of the axis without experiencing a traumatic episode. Plain radiograph and CT image showed 7.0 mm anterolisthesis of the axis. Both C2 and C3 facet joints positioned asymmetrically, and the unilateral side oriented coronally, which was less resistant to rotational motion. These facet joint abnormalities could cause segmental instability and spondylolisthesis of the axis. Due to the resultant myelopathy, the slip with cord compression was surgically corrected by posterior decompression with instrumented fusion.

## 1. Introduction

Hangman's fracture is known as traumatic spondylolisthesis of the axis. It occurs due to a bilateral fracture of the C2 pars interarticularis. This fracture is typically a result of high-energy trauma and hyperextension [1, 2]. It accounts for 20 to 22% of all axis fractures [3]. Reports on hangman's fracture frequently appear in publications. However, there are very few reports describing spondylolisthesis of the axis without a traumatic event. In this report, we present a rare case of a 49-year-old man with nontraumatic spondylolisthesis of the axis.

## 2. Case Presentation

**Figure 1:**
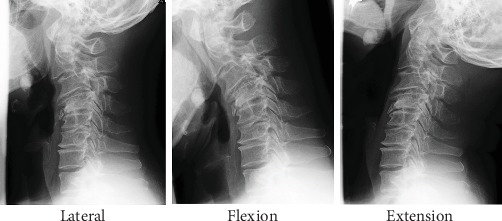
Preoperative X-ray. Axis slipped anteriorly by 7 mm. No change in slippage was observed in the flexion and extension positions.

**Figure 2:**
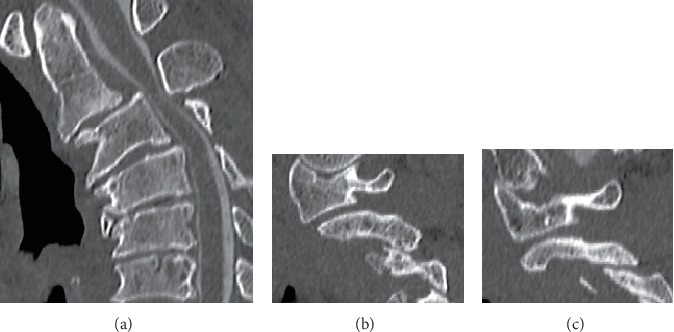
Preoperative CT myelography images. (a) Sagittal view. (b) Right view and (c) Left view of the C2-3 facet joint. CT images show that the bilateral pars interarticularis were not fractured.

**Figure 3:**
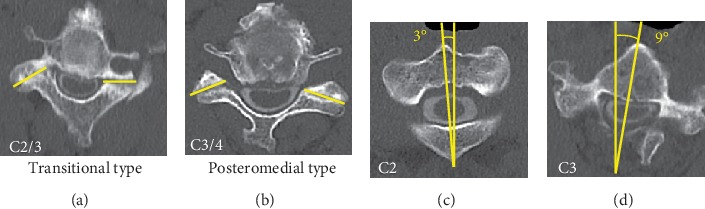
(a) The facet joint of C2/3 was transitional. (b) The facet joint of C3/4 was posteromedial, (c, d) C2 vertebra rotated to the right and to the lower vertebra.

**Figure 4:**
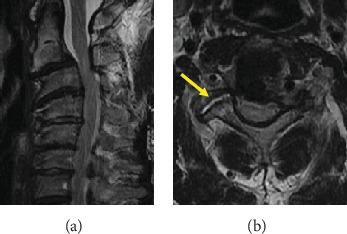
Preoperative MRI images. (a) Preoperative MRI images showed spinal cord compression at C2-3 level with spinal edema. (b) Axial MRI images of C2-3 right facet joint indicate an articular edema.

A 49-year-old man was referred to our clinic, complaining of bilateral numbness in the hands, a disorder affecting hand dexterity and gait disturbance. He had no history of any traumatic accidents or such, including in his childhood. He was an agricultural engineer and had neither comorbidities nor the history of notable sport activities. The neurological examination revealed bilateral-hand muscle weakness and hypesthesia. Plain radiograph showed a marked case of spondylolisthesis of the axis. The axis had slipped 7 mm anteriorly. There was no slippage reduction in the flexion and extension positions according to the radiographs (Figure 1). We observed cervical kyphosis with -20 degrees on the C2-7 angles. CT images revealed no fractures at the pars interarticularis indicating hangman's fracture (Figure 2). The C2-3 facet joints were spatially asymmetrical and coronally oriented on the left side. Furthermore, the C2 vertebra had rotated clockwise compared to the lower level (Figure 3). By contrast, C3-4 facet joints were symmetrical, and both facing C3-4 facet joints formed a bowl-shaped plane, which was resistant to rotational movement. MRI images demonstrated spinal cord compression at the C2-3 level with spinal cord edema, the following multilevel cervical spinal cord decompression, and facet joint edema at C2-3 (Figure 4).

**Figure 5:**
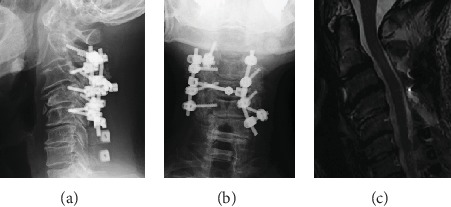
(a, b) Postoperative X-ray. X-ray shows C2-5 posterior fusion. Anterior spondylolisthesis of the axis was corrected. (c) Postoperative MRI image. MRI image shows that decompression had been performed and that the central cord deficit had been improved.

We performed C2-5 posterior decompression with C2 partial laminectomy and C3-5 laminectomy and C6-7 laminoplasty for progressive myelopathy. Then, we inserted the pedicle screws as possible for strong fixation except for left C3 and C4 that were inserted lateral mass screws for instrumented fusion. The anterior slip and rotational deformity of axis were fully corrected (Figure 5). Cervical kyphosis was corrected from -20 degrees to -15 degrees. Bilateral numbness in the hands and the disorder affecting hand dexterity were completely resolved. The JOA score improved from its preoperative score of 11.5 to its postoperative score of 17. Two years following surgery, the corrected alignment had been maintained without any instability. We observed no symptoms of any recurrence.

## 3. Discussion

Traumatic spondylolisthesis of the axis is a failure in the pars interarticularis of the neural arch and a separation from the C2 vertebral body [4]. On the other hand, the main cause of degenerative cervical spondylolisthesis is arthrosis of the facet joints, disc degeneration, and cervical sagittal malalignment [5]. Cervical spondylolisthesis is not a rare condition in the elderly. The most frequently involved levels were C3/4 (46%) and C4/5 (49.4%). In contrast, the cervical spondylolisthesis occurring at C2/3 was reported to less frequent (6.8%) [6]. We presented a 49-year-old patient with nontraumatic spondylolisthesis of the axis and cervical kyphosis. The presented case had the morphological asymmetry of C2-3 facet joints in addition to degenerative changes including facet joint arthrosis, disc degeneration, and kyphotic deformity. Takano et al. suggested that thinning of the facets and narrowing of the joint space may be the primary causes of degenerative cervical spondylolisthesis rather than disc involvement [7]. Furthermore, Pal and colleagues analyzed the orientation of the superior articular surfaces from C3 to T3 using axial CT slices and categorized orientation of the facet joints into posteromedial, posterolateral, and transitional types [8]. All C2-3 facet joints were reported to show posteromedially facing superior articular facets [8–10]. In this case, the C2-3 facet joint showed transitional type and posteromedial type on the left and right sides, respectively. Rong suggested posteromedially oriented facet joints could restrict the axial rotation by the contralateral facet, acting as a barricade. The posteromedially oriented facet joints at C2-3 level thus were of significant importance for the stabilization of the C2 vertebra during rotational movement, facilitating the C1-2 rotation [10]. The transitionally oriented facets restrict rotation and lateral bending only on one side, by both superior articular facets facing in the same direction. This suggests that morphological abnormalities at the C2-3 facet joint in our current case may have affected rotational stability. In addition, the C2-3 facet joint had articular edema and vertebral rotation compared to the lower level. These findings also indicate a segmental instability at the C2-3 level.

In conclusion, we have presented a rare case of nontraumatic spondylolisthesis of the axis, which is relevant to facet joint pathology. The abnormal orientation and asymmetry of the C2-3 facet joints points to segmental instability, which lead to spondylolisthesis of the axis in the absence of a traumatic event.
